# Toner Waste Powder (TWP) as a Filler for Polymer Blends (LDPE/HIPS) for Enhanced Electrical Conductivity

**DOI:** 10.3390/ma12193062

**Published:** 2019-09-20

**Authors:** Salim Hammani, Ahmed Barhoum, Sakthivel Nagarajan, Mikhael Bechelany

**Affiliations:** 1 Laboratoire d’Analyse Fonctionnelle des Procédés Chimiques, Faculté des Sciences de l’ingénieur, Université BLIDA1, BLIDA B.P 270, ALGERIE; hammani71@yahoo.fr; 2 Institut Europeen des Membranes, IEM – UMR 5635, ENSCM, CNRS, Univ Montpellier, Montpellier 34090, France; sakthi.20@hotmail.com; 3 Chemistry Department, Faculty of Science, Helwan University, Ain Helwan, Cairo 11795, Egypt

**Keywords:** toner waste powder, composite, electrical conductivity

## Abstract

Rapid urbanization proportionally increases the waste products which force humankind to find a suitable waste management system. This study aims at identifying the possibility of using toner waste powder (TWP) as a filler for fabricating polymer composites for enhanced electrical conductivity of polymer blends. TWP was successfully incorporated into a polymer blend of low-density polyethylene/high impact polystyrene (LDPE/HIPS) at a high loading percentage of up to 20 wt %. Elemental analysis (SEM-EDS and XRF) showed that the main constituents of TWP are carbon and iron with traces of other metals such as Ca, Cs, Ti, Mn, Si. The electrical conductivity of LDPE/HIPS is significantly enhanced by loading the TWP into the polymer blend. The addition of TWP to LDPE/HIPS blend decreases the electrical resistivity of the LDPE/HIPS/TWP composite to ~2.9 × 10^7^ Ohm.cm at 10 wt % of TWP, which is several orders of magnitude lower than that of the neat blend with maintaining the thermal stability of the polymer composite. The prepared polymer composite is lightweight and shows electrical conductivity, thus it can have potential applications in electronic materials and automotive industries.

## 1. Introduction

Industrial wastes are considered to be one of the important raw materials for manufacturing waste-based products. Various wastes such as industrial waste, municipal waste, hazardous waste including biomedical and clinical waste, special hazardous waste including radioactive waste, explosive waste, and waste from electrical and electronic equipment are widespread [1]. According to IMARC Group, the global ink market has reached a value of US $ 19.6 Billion in 2018, growing at compound annual growth rate (CAGR) of 3.6% during 2011–2018. The CAGR value will decrease between 2018 and 2023 by about 3.2% to reach US $ 23 billion in 2023 [2]. The increased global demand for ink production has resulted in high accumulation of their wastes and is a threat to the environment. Utilization of the ink wastes for the fabrication of materials can be a promising approach in ink waste management. It can also support the economic sector. 

The recovery and reuse sector of the industrial waste in the preparation of polymer composites is booming. Various studies proved that waste materials can be employed for the fabrication of functional material. For example, Hassan et al. [3] used the eggshell-based CaCO_3_ as a filler to prepare biopolymer composites. The incorporation of 2% eggshell filler in the bioplastic increases the thermal and mechanical properties of the obtained composite. Addul Khalil et al. [4] have incorporated a bio-agricultural waste as a filler in the preparation of oil palm ash (OPA)/epoxy composites. The incorporation of OPA increased the density, thermal stability, tensile and flexural properties of the prepared composites. Waste tire dust (WTD)/carbon black (CB) hybrid was used as a filler in natural rubber (NR); the increment of CB to WTD in the composite increased the tensile properties such as tensile strength, elongation break, and tensile modulus [5]. 

The preparation of polymer composite using an immiscible polymer blend is one of the important methods to enhance the interfacial adhesion and the percolation threshold of the filler particles. The polymer nanocomposites based on conducting nanofillers (e.g. graphene, carbon nanotubes, carbon nanofibers) have found widespread application in industrial applications [6,7,8,9,10,11,12,13]. The introduction of nanofillers in immiscible polymer blends enhanced several properties such as thermal properties, conductive, barrier and electrical properties [10,11]. Yang et al. [10] enhanced the conductive and the impact strength by introducing the carbon black (CB) in the immiscible blend (PP/EPDM). The incorporation of TiO_2_ in PET/LDPE enhanced the dispersion of LDPE in the PET and improved the thermal stability and permeability of the film for water vapor [11]. LDPE/HIPS is an immiscible polymer blend and has a weak interaction between the two phases, the incorporation of filler nanoparticles increased the interaction and the mechanical properties [14,15]. Recovery of industrial waste is the most appropriate way to protect the environment. Several studies have been conducted in this direction [16,17,18]. Recovery and valorization of ink are necessary because of its classification as a hazardous waste product [16]. The recovery of the TWP can reach 80% according to the study conducted by Lu et al. [17].

The main constituencies of the conventional inks are pigments, carriers (vehicles), binders, and additives. The color and opacity to the ink are obtained using organic or inorganic pigments which also influence the ink fluidity. Low molecular weight polymeric resins are used as binders which facilitate the dispersion of the pigments and supports to retain the ink on the plastic film surface after printing. The liquid carrier (or vehicle) provides fluidity to the ink and transfers the ink from the printing system to the substrate [19]. 

Production of high-performance polymer materials at a low cost remains a challenging issue from an industrial point of view. The recycling of TWP is the best way to protect the environment. It is worth to mention that the landfill and incineration of waste toner cartridges are not ecofriendly for the following reasons: (i) The cartridge plastics have a very slow decomposing rate; (ii) the incineration of the waste toner cartridges may produce furan and dioxin gases; and (iii) the toner waste powder will leak out so as to pollute the environment during landfill or incineration process [20]. The re-utilization of TWP as a filler in the polymer composites and the enhancing of the physicochemical properties of polymers using TWP are valuable approaches in ink waste management. TWP is mainly produced from the disposed toner cartridges. The small size (2–7 μm) particles and components of polystyrene, polyacrylate, Fe_3_O_4_, and SiO_2_ make TWP a hazardous material. It is also found that inhalation of TWP can cause blood and stomach cancer. However, to date, little information has been published about the disposal technology of waste toner.

This work explores an environmentally friendly technology for the disposal of the waste toner i.e., by using TWP as a filler for the fabrication of polymer composites. Different characterization techniques such as Fourier transform infrared spectrometry (FTIR), scanning electron microscopy (SEM) connected with energy-dispersive X–ray spectroscopy (EDS–SEM), X-ray fluorescence spectroscopy (XRF) were used to examine the chemical composition of TWP [21,22]. The discrimination and the identification of the elements in the TWP provide the information to understand the possibility to employ TWP as a filler for polymers. For this reason, a combination of techniques typically used for the analysis of ink is chosen. The prepared TWP/LDPE80/HIPS20 composite films with different amounts of TWP were analyzed by thermogravimetry analysis (TGA) and differential scanning calorimetry (DSC). The electrical conductivity was also measured. The polymer component typically has structural functions and processability, whereas the TWP can introduce specific functionalities such as enhanced electrical conductivity and magnetism as well as improved mechanical and thermal properties.

## 2. Materials and Methods 

### 2.1. Materials

High impact polystyrene (HIPS LADENE330), manufactured by SABIC (Kingdom of Saudi Arabia), with a melt flow index IF = 4 g/10 min and average molecular weight (M_w_) of 211760 g/mol and density of 1.04 g/cm^3^ was used as the dispersed phase. The matrix is a low-density polyethylene (LDPE HP0322N) manufactured by SABIC (KSA), with a melt index IF = 0.33 g/10 min and a melting point T_f_ = 190 °C. The nanofiller used is TWP. It was recovered from the printer-copier of our university. This type of ink is sold by Colorpoint Technology (New Taipei City, Taiwan). Tetrahydrofuran (C_4_H_8_O, 99.9%, Sigma-Aldrich, Taufkirchen, Germany) and xylene (C_6_H_4_(CH_3_)_2_, 98.5%, Sigma-Aldrich, Taufkirchen, Germany) were used as solvents. 

### 2.2. Composite Films Preparation

HIPS (0.03 g) were dissolved by using 10 mL tetrahydrofuran (THF) as the solvent by magnetic stirring at room temperature. Under stirring and in a reflux assembly, the LDPE (0.12 g) solution was prepared using 10 mL xylene as the solvent at room temperature, until a homogeneous dispersion is obtained. After the dissolution of the two polymers, different loadings of TWP (2, 6, 10, 15, and 20 wt %) with respect to the weight of LDPE/HIPS were incorporated to the LDPE/HIPS/TWP solutions. After completely wetting the TWP with the polymer blend, mechanical stirring (180 rpm, 60 min) was performed to disperse the TWP in the polymer mixture. The polymer composites (LDPE/HIPS/TWP) were precipitated and dried in the oven at 60 °C for 24 h to eliminate the residue solvent. A blend without adding TWP was also prepared following the same procedure. The thickness of the composite films obtained was about 25 ± 2 μm. The composites films were prepared with different amounts of TWP by mixing a weight of TWP filler (*w_f_*) in weight of polymer blend (*w_p_*). Hence the percentage weight fraction (%) of the TWP filler is calculated by the following equation:

(1) $$f\left(\%\right)=\frac{w_{f}}{w_{p}+w_{f}}.100$$

The prepared compositions are illustrated in Table 1.

### 2.3. Characterization Techniques

The characterization was carried out in two parts: first, only the TWP was characterized, then and in the second part, we focused on the characterization of the polymer composite films (LDPE/HIPS/TWP) with different TWP loadings. 

All the characterization techniques were performed after drying the TWP for 1 h at 80 °C and finely powdered. X-ray powder diffraction (XRD, BRUKER, Mannhein, Germany) analysis of the TWP was performed with a D2 PHASER BRUKER diffractometer XRD, (BRUKER, Mannhein, Germany) using CuK_α_ radiation (40 KV and 30 mA) with a wavelength of 1.5418 A and scanning speed of 0.5°. X-ray fluorescence spectrometer (XRF, E-pislon3–XL; Pan Analytical Corp. (Brevannes, France) and energy dispersive microanalysis (EDS, Oxford instrument EDS Aztec, Wiesbaden, UK) were used to investigate the elemental analysis of this powder. The surface morphology of the TWP was studied by field emission scanning electron microscopy (FESEM, ZEISS, EVO HD15, Oberkochen, Germany). The surface morphology of the composites was analyzed by a scanning electron microscope (SEM, Jeol JSM–6360LV, Jeol, Tokyo, Japan). FTIR spectra of TWP and composites were conducted on a Fourier transform infrared spectrometer (FTIR 4100, Perkin-Elmer Company, (Waltham, MA, USA) using KBr pellets analysis. The FTIR spectra were recorded ranging from 400 to 4000 cm^−1^ at 4 cm^−1^ resolution.

Differential scanning calorimetry measurements were carried out on the DSC Q20 TA Instrument (TA, New Castle, DE, USA). Films with weights ranging from 2 to 3 mg were used. The composite films were heated up to 180 °C and cooled down to 60 °C. The films were annealed at 180 °C for 15 min with a heating/cooling rate of 10 °C/min. TGA method was used for the thermal stability analysis of the polymer composite. Thermogravimetric measurements of the composites LDPE/HIPS/TWP were conducted on TA Instrument Q500 TGA (TA, New Castle, DE, USA) thermobalance at heating rates of 10 °C/min in a temperature range from 60 to 700 °C under a steady flow of air atmosphere (60 ml/min). The composite films weighing approximately 15 mg were used for the analysis. The DC electrical conductivity of composite films was determined by two probes technique using a digital multimeter (GW Instek GDM-8255A, CA, USA) equipment interfaced with a desktop PC. The standard deviation is calculated based on at least three measurements.

## 3. Results

The results part is divided into two sections. The first section is dedicated to the examination of the composition of TWP. The second one discusses the effect of adding TWP on the morphology, thermal, degradation, and electrical properties of LDPE/HIPS/TWP composites.

### 3.1. Toner Waste Powder 

#### 3.1.1. Morphology and Elemental Analysis of Toner Waste Powder 

**Figure 1:**
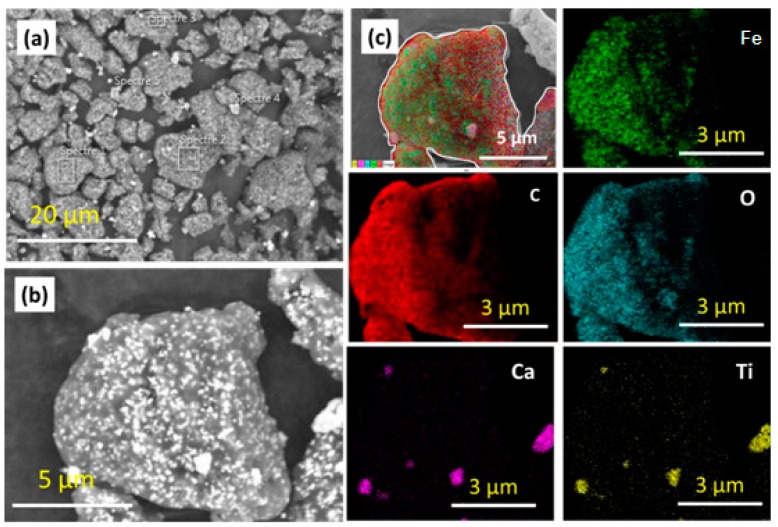
Scanning electron microscopy (SEM) image and energy dispersive x-ray map of TWP: (**a**) low-magnification SEM image; (**b**) high-magnification SEM image; and (**c**) energy-dispersive X–ray spectroscopy (EDS)-elemental mapping of TWP particles.

**Figure 2:**
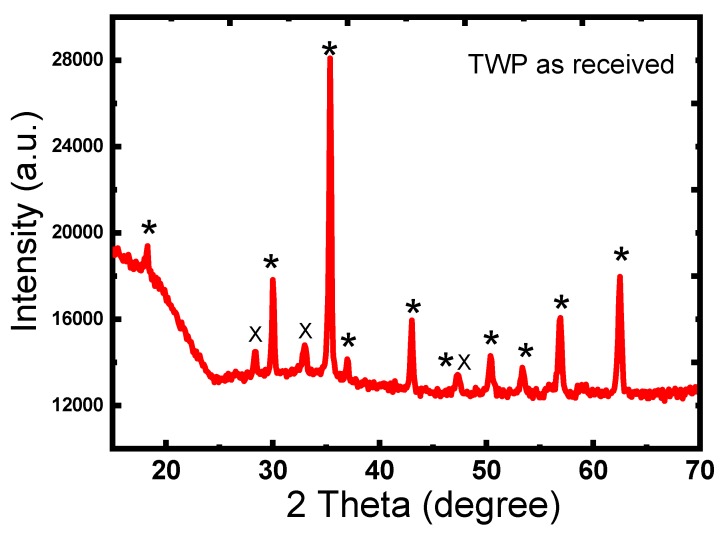
XRD of TWP after drying in air atmosphere at 80 °C for 1 hr: Fe_2_O_3_ (*) and CeO_2_ (x).

The TWP morphology was analyzed by SEM microscopy and their elemental composition was investigated by EDS technique at four different positions of the TWP sample, as shown in Figure 1. The particles in Figure 2 have an irregular and particle morphology with an average size of 100 to 500 nm. The EDS elemental analysis presents a high diversity of the composition at different positions (Table 2). The C, O, Fe, Si, and Ca elements cannot be practically detected in all the positions. The analysis of the EDS results shows that carbon (in red) is the most abundant element in this composition with oxygen (Figure 1C). The signal of carbon should mainly originate from the polymer phase or the carbon tab that has been used on the top of SEM sample holder. Iron is the most important element compared to other mineral elements. 

The TWP was characterized by XRF to accurately determine the elemental composition. Typically, XRF and EDS are very close techniques. However, XRF is a more accurate technique than EDS because of the fact that: (i) XRF is always performed with certified standards whereas EDS analysis is typically done without any standards; (ii) XRF has a detection limit at the ppm level, while SEM-EDS detection limit is typically at 0.1% level depending on the accelerating voltages applied; and (iii) the XRF irradiation area is larger than that of EDS. For inhomogeneous samples such as TWP, the XRF may detect extra elements compared to EDS. The XRF analysis of the TWP shows that Ti, Ca, and Ce constitute only a few percent of the overall composition, which has been confirmed by the XRF results. The coupling of XRF and SEM-EDS techniques for the identification of the TWP gives the most important synergic effect [23,24]. X-ray analysis providing information about pigment enables one to discriminate between TWP components [25]. Table 3 shows that the TWP contained Fe, Ti, Ca, and Ce as the main components and Si, P, Zn, and Mn as trace elements [20,24,25].

#### 3.1.2. Crystallinity and Crystal Structure of Toner Waste Powder 

Figure 2 shows the XRD patterns of the TWP as received. All diffraction peaks were centered at 18.3°, 30.1°, 35.4°, 37.0°, 43.0°,47.3°, 53,4°, 57.0°, and 62.5°, corresponding to (111), (220), (311), (222), (400), (331), (422), (511), and (440) crystallographic planes of the crystalline α-Fe_3_O_4_ (magnetite) [26]. The sharp characteristic peaks detected reveal the high crystallinity of Fe_3_O_4_ in TWP. The peaks at 28.5°, 33.0°, and 47.5° could be indexed to the diffraction peaks of (111), (200), (220) crystal planes of CeO_2_ particles respectively [27]. It is highly probable that there is an overlap between the peaks of Fe_2_O_3_ with CeO_2_ (47°) and another overlap with TiO_2_ at (35°) [28]. The average crystallite sizes of the TWP were determined using the Debye– Scherrer equation (Equation (2)).

(2) D=0.9λ/β cosθ

where λ is the wavelength of the X-ray (nm); β is the full width at half maximum (FWHM), and θ is the Bragg diffraction angle. The average crystallite size of Fe_2_O_3_ calculated from the full width at half-maximum of the peak at 2θ = 35.4° using Scherrer equation is about 38 nm.

### 3.2. TWP/Polymer Composites

#### 3.2.1. FTIR of the TWP and TWP/Polymer Composites

**Figure 3:**
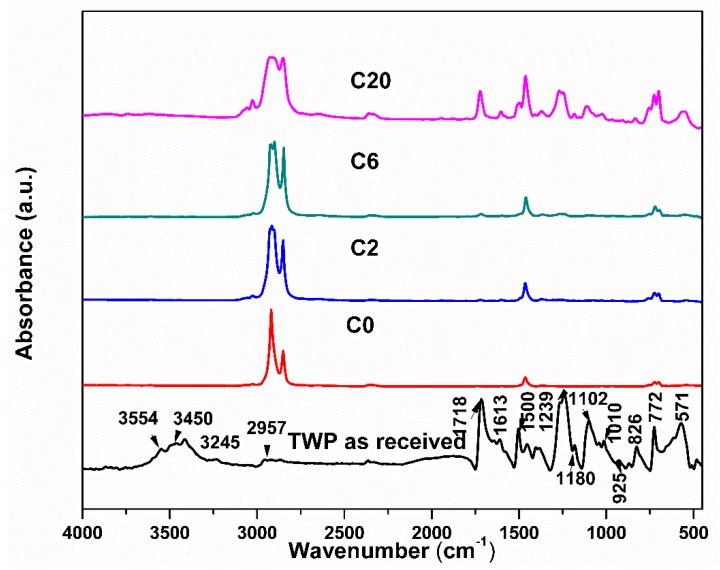
Fourier transform infrared (FTIR) spectrum of the TWP and composite samples.

The FTIR spectrum for pure blend and composites is shown in Figure 3. FTIR confirms the interaction between the polymers and TWP over wavenumber range of 450–4000 cm^−1^. Figure 3 shows the characteristic peaks for LDPE/HIPS at 3024 cm^−1^ that correspond to the C–H bond stretching vibrations of the benzene ring [29]. The peaks at 2900 and 2850 cm^−1^ are attributed to asymmetric and symmetric –CH_2_– respectively. The characteristic peaks of LDPE/HIPS were positioned at 1460 and 723 cm^−1^ which attributed to the spectral vibration of (–CH_2_–) scissoring and rocking, respectively. The peak at 723 cm^−1^ is the internal reference of the LDPE (and remains unchanged with loading TWP). The addition of TWP in the LDPE/HIPS blend shows that all the characteristic peaks of the blend existed. All the remaining peaks on the FT–IR spectrum of the composite are attributed to the TWP. The main ingredients of the conventional inks are pigments (organic and inorganic), binders (polymer), carriers, and additives [19]. The FT–IR spectrum of the TWP located between 3700 and 3300 cm^−1^ corresponds to O–H and N–H stretching vibrations. The band around 2995 cm^−1^ represents stretching vibrations of C–H for a CH_3_ group. As it is well-known, carbonyl compounds have a strong IR absorption at 1700–1800 cm^−1^. The peaks at 1713 and 1240 cm^−1^ are assigned to carbonyl compounds [29]. The peaks at 1460 and 726 cm^−1^ are assigned to methylene (–CH_2_–) [29], and the weak peaks at 1613 to stretching vibration for carbonyl group (C=C). In this case, broadband at 1090 cm^−1^ was observed and assigned to C–N vibrations coming from amine molecules. The strong band at 570 cm^−1^ belongs to the stretching vibration mode of Fe–O bonds in Fe_3_O_4_ [30,31]. The strong absorption peak around 450 cm^−1^ is attributed to the vibration of Ti–O–Ti bonds in TiO_2_ [32,33]. 

#### 3.2.2. Polymer Composites Morphology

**Figure 4:**
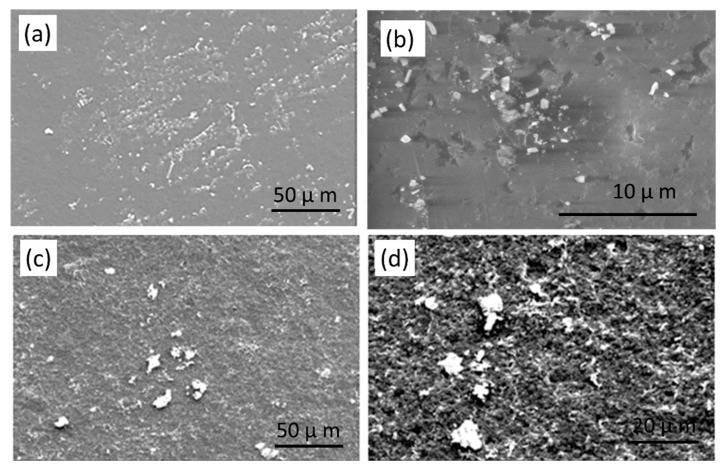
Low and high magnification SEM images of LDPE/HIPS composites loaded with TWP: (**a**,**b**) 2 wt %; and (**c**,**d**) 10 wt %.

Since the developed morphology and dispersity can strongly influence the electrical properties of composites, it is important to understand clearly the basic mechanisms of morphology development [6,7,8,9,10,11,12,13]. The size and morphology of the TWP particles in the polymer composites were assessed with SEM. The surface morphologies of composites with 2 and 10 wt % of TWP are presented in Figure 4. At 2 wt % of TWP, the filler of TWP particles are well-dispersed in the composite. At 10 wt % TWP, the presence of the aggregates is noted (Figure 4c,d), this may be due to the high amount and strong surface interaction of the filler in the composite [34,35]. 

**Figure 5:**
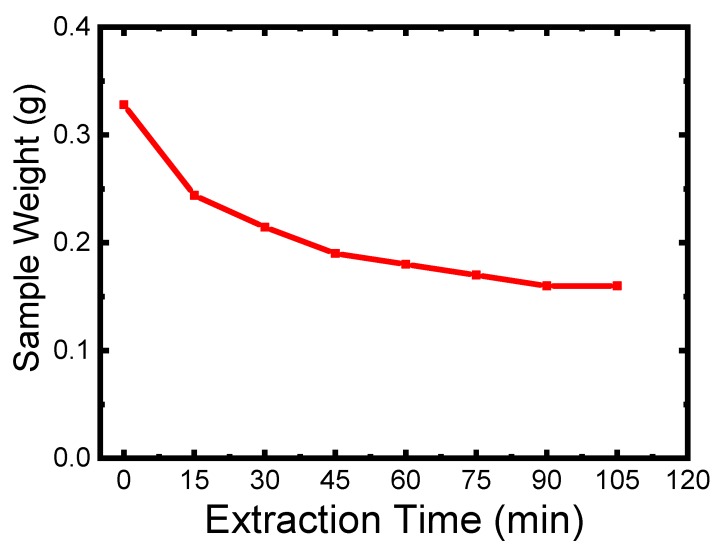
Variation of polymer composite sample weight as the function of time.

The distribution of TWP may have an effect on the electrical conductivity and thermal stability of the prepared polymer composite. LDPE and HIPS are the two immiscible polymers, so it is worth to mention that, there are two possible ways of TWP dispersion in the polymer blend. The first way is that the TWP is predominantly dispersed in one phase of the polymer blend (LDPE or HIPS). The second way is the dispersion of the TWP at the interface of the two polymers. The interfacial energy of the two polymers is the main factor determining the uneven dispersion of TWP in polymer blend. In this context, polymer composite was prepared with the following composition, LDPE (50 wt %)/HIPS (25 wt %)/TWP (25 wt %). The sample LDPE/HIPS/TWP25 was immersed in the solution solvent (THF) in order to extract the HIPS phase. Figure 5 shows the variation of the weight of the LDPE/HIPS/TWP25 after each extraction-drying. It has been noted that the kinetics of extraction is fast up to 45 min, and then the extraction stabilizes to obtain a constant weight. According to the curve (Figure 5), the sample weight is reduced by almost 50 wt % and the color of the sample becomes white. This may indicate that the black TWP is mainly dispersed within the HIPS phase. The study conducted by Sangroniz et al. [10] showed that the TiO_2_ NPs were located at the interface between PET80/LDPE20 immiscible polymer blend. 

#### 3.2.3. Differential Scanning Calorimetry (DSC)

**Figure 6:**
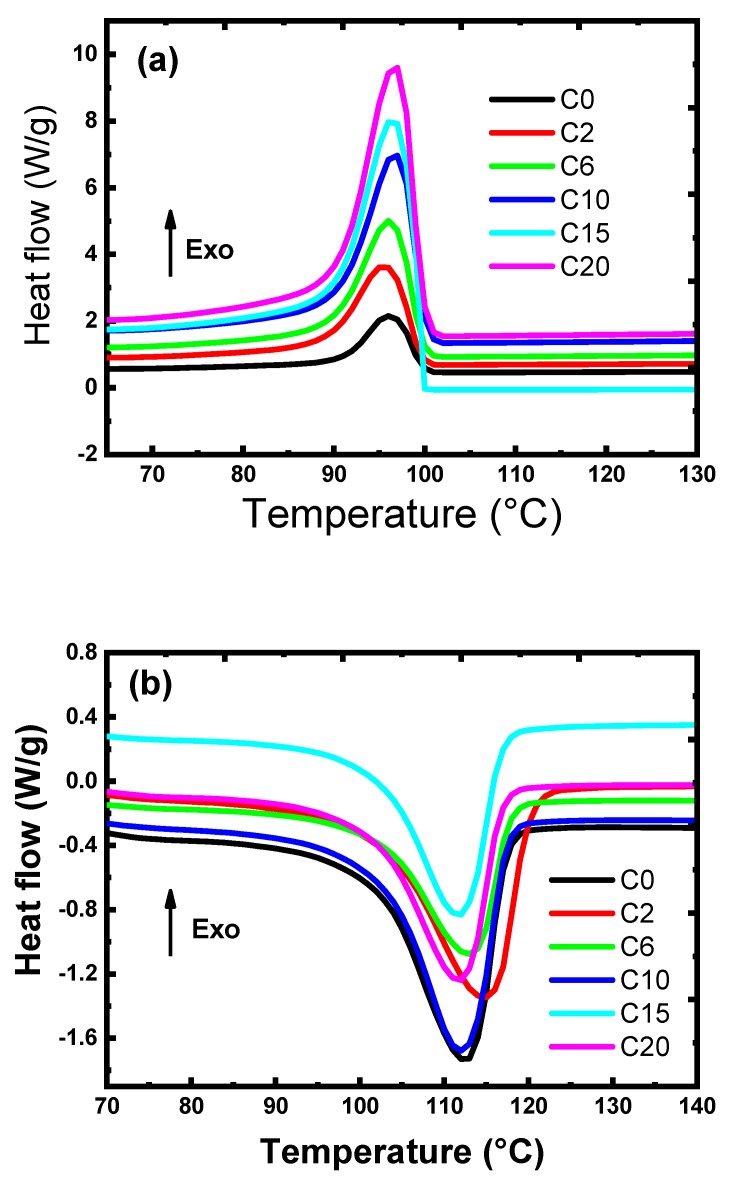
Differential scanning calorimetry (DSC) curves of LDPE/HIPS/TWP polymer composites: (**a**) cooling curve showing the crystallization peak; and (**b**) heating curve showing the melting peak.

The differential scanning calorimetry (DSC) can be used to investigate the thermal transition of the blend (LDPE/HIPS) and TWP. Crystallization exotherms and melting endotherms were recorded on DSC measurements and are shown in Figure 6. The degree of crystallinity ($\chi_{c}$) of LDPE/HIPS and its composites were calculated from DSC endotherms curves according to the Equation (3).

(3) $$\chi_{c}=\frac{\Delta H}{{\Delta H}_{m}\;.\;W_{\mathrm{LDPE}}}$$

where ΔH is the heat of fusion for the polymer composite, ${\Delta H}_{m}$ is the heat of fusion for completely crystallized LDPE (293 J/g) [36], and W_LDPE_ is the weight fraction of the polymer matrix.

From Table 4, the crystallization and melting temperatures of LDPE/HIPS are about 96.6 °C and 112.1 °C respectively. These two values increase slightly with the increase of the TWP load, up to 10 wt % and then begin to decrease until they reach 98 °C and 114.5 °C. Loading TWP into the composite has an irregular increase effect on LDPE/HIPS crystallinity and melting temperatures. However, the addition of TWP leads to a significant decrease in the melting and crystallization enthalpies of the prepared composites (Table 4). The melting enthalpy decreased from 90.9 J/g to 76.0 J/g and the crystallization enthalpy decreases from 66.5 J/g to 47.4 J/g with increasing the TWP loading up to 20%. Moreover, adding TWP in LDPE/HIPS up to 20 wt % decreases the degree of crystallinity in the composites from 25.4 to 18.1%. This may be due to that the TWP particles play the role of a heterogeneous nucleating agent to improve the crystallinity level of the LDPE in the composites [36]. TWP can hinder the transport of molecule chains to reduce the degree of crystallinity; this phenomenon may be due to the heterogeneous distribution of the different elements in TWP and the composition of the TWP (as given in Table 3). TWP can be also dispersed into the amorphous regions and prevents the nucleation and growth of the crystalline domain and thereby reduces the degree of crystallinity [37]. These results are in good agreement with those reported by Ahangaran et al. [30].

#### 3.2.4. Thermogravimetric Analysis

**Figure 7:**
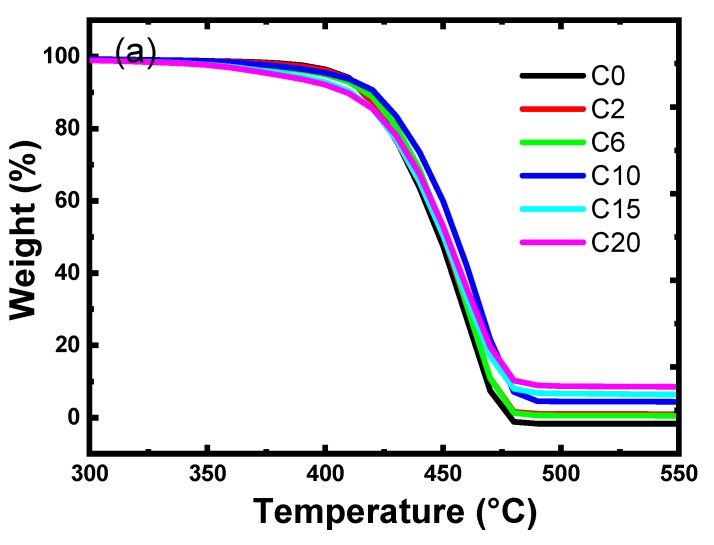

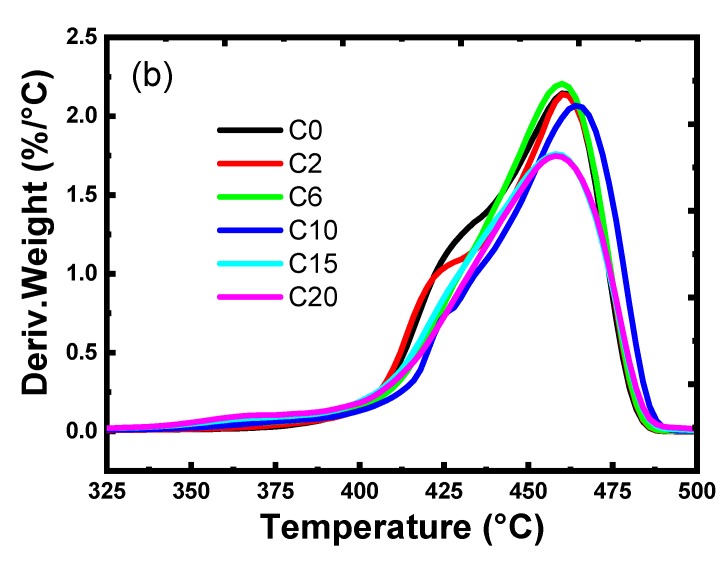
Thermograms of pure LDPE80/HIPS20 and of the different concentrations (2, 6, 10, 15, 20 wt %) of TWP loaded LDPE/HIPS/TWP composite films: (**a**) TGA; and (**b**) DTG.

The thermal stability of polymer composites could begin with oxidative processes that are accelerated under elevated temperatures. There are several factors affecting the thermal stability of the polymer composites i.e., average molecular weight of the polymer, presence of weak linkages or irregular structures such as such as branches, functional groups, or unsaturation, and presence of filler particles. Thermal stability of the prepared composites was investigated by thermogravimetric analysis [31,32,36,37]. The thermogravimetry curves (TGA) (% mass/degradation temperature) and corresponding derivative thermogravimetric (dTGA) curves (mass loss rate in function of temperature) of LDPE/HIPS/TWP composites obtained at 10°C/min are shown in Figure 7a,b respectively. In Figure 7a, the TGA analysis for the composites was performed in the temperature range from 60 to 700 °C. The TGA curves show that the polymer and the composites display a similar degradation profile. Analysis data from curves (tabulated in Table 5) were used to assess the effect of TWP content on the stability and the degradability pattern of LDPE/HIPS. The degradation of polymer composites starts at 340 °C and finishes at around 580 °C. The loss fraction was assumed to be a measurement of thermal stability, the degradation temperature slightly increases from 417 °C to 422 °C when the TWP loading in composite increases from 2 to 10 wt %. Above 15% of TWP, the thermal stability of the composite slightly decreases to attain 409 °C at 20% of the TWP. Thus, the initial decomposition temperatures at about 10% mass loss are given in Table 5. The DTG of the polymer composites shows a shoulder on the curves, which shows the presence of two degradation rates. The shoulder on the DTG curves shifts to the left from 420 to 370 °C with the increase of the TWP in the composites from 0 to 10 wt %. This shows that the rate of thermal degradation within the composites is slightly accelerated as the TWP content increases in the polymer blend. The char residue calculated from the TGA curves is given in Table 5. The improvement in the thermal stability of polymer composites is due to the formation of polymer–filler network by physical cross-linking of polymer chains through the filler particles, which restrict the thermal motions of polymer chains [38,39]. However, the slight increase or decrease in the thermal stability could be linked to the fact that some filler particles catalyzed oxidative degradation processes. In this context, the slight improvement in the thermal stability of the prepared composite might be due to a catalyzed oxidative degradation process of the TWP particles. 

#### 3.2.5. Electrical Resistivity

The electrical conductivity of the polymeric materials is the degree to which these materials conduct the electricity. Electrical resistivity of the polymeric materials is the degree to which these materials oppose the flow of electricity. Thus, the polymeric material with low electrical resistivity indicates the high electrical conductivity of the material. Polymer materials by themselves have high electrical resistivity and are, thus, electrical insulators. In general, conductive polymer composites are mainly composed of insulating polymer matrices and conductive fillers with high conductivity, in which conductive fillers provide carriers. The most frequently used conductive fillers are carbon black, carbon fibers, metal powders, and graphene [40,41,42,43]. The charge carriers transfer into polymer composites by the interaction between the filler particles. The synergic effect on the composite’s conductivity is related to the use of more than one filler. The increase in filler content to a certain level initiates the formation of filler network in the polymer. A optimum filler content can increase the electrical conductivity of the polymer nanocomposite by several orders of magnitude. This sudden increase in the electrical conductivity is called percolation threshold. A good selection of filler can result in better contact between the filler particles and prolonged non-interrupted conduction paths. The characteristics of fillers (composition, size, shape, orientation/dispersibility in the polymer) play a significant role in revealing the percolation properties [40,41]. Fillers with a higher aspect ratio, such as graphene nanosheets and carbon nanotubes, have shown better electrical conductivity than the carbon black of spherical particle morphology. However, from economic factors, the research into recycling of TWP and reusing it as a conductive filler in polymers seems to be the matter of particular interest.

**Figure 8:**
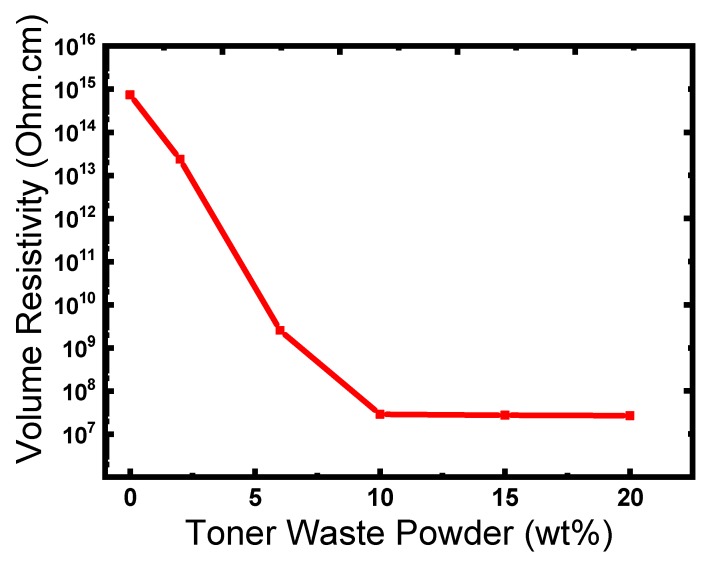
Volume resistivity variation as function of TWP loaded in LDPE/HIPS/TWP composites.

The electrical resistivity is a so-called specific electrical resistance, bulk resistivity, specific volume resistance, or simply resistivity. The electrical resistivity can be used to produce conductor or insulator polymeric materials for a specific application. If the electrical resistivity is below 10^5^ Ohm.cm the polymeric material can be considered as conductive and if it is above 10^9^ Ohm.cm the material become an electrical insulator. Electrical resistivity in the produced polymer composites depends on the filler (aspect ratio and content), filler dispersibility, as well as the polymer composite microstructure. In this study, the surface resistivity was investigated for different composites and the results were plotted as a function of TWP loading (Figure 8). As the TWP content increases, the surface resistivity decreases, this may be due to the establishment of a 3D network of TWP in the LDPE/HIPS/TWP composite. A sharp decrease of electrical resistivity at 6 wt % TWP content is noted from Figure 8 implying that a conductive network was formed in the composite. For LDPE/HIPS/TWP composites with 10 wt % TWP, an electrical resistivity value of around 2.90 × 10^7^ Ohm.cm was obtained, which is several orders of magnitude lower than that of the blend polymer LDPE/HIPS. The electrical percolation threshold is reached at approximately 10 wt % of TWP. In similar work, Liang et al. [11] prepared the low-density polyethylene/CNT nanocomposites via melt blending and they reported low CNT percolation threshold content of about 3.6 vol %. Luyt et al. [44] loaded low-density polyethylene (LDPE) and linear low-density polyethylene (LLDPE) with different copper powder contents using the melt mixing technique. The percolation threshold is reached at 18.7 vol % for both LDPE and LLDPE composites. Van der Schoot’s [42,43] performed studies to predict that the addition of a small fraction of longer rods to a polymer system can significantly lower the threshold, and this effect is stronger for a larger length of the rods. 

## 4. Conclusions

The present work aims at identifying the possibility of using TWP as a filler for producing polymer composites with enhanced electrical conductivity and maintaining the thermal stability of the polymer composite. The elemental composition, crystalline structure, particle size, and morphology of TWP were investigated by XRD, FTIR, XRF, and SEM–EDS techniques. The XRF results show the presence of Fe, Ti, Ca, and Ce as the majority element and Si, Zn, Mn, and P as traces. Furthermore, XRD analysis confirmed the molecular state of Fe in TWP as crystalline Fe_3_O_4._ DSC measurements showed a slight increase of crystallinity and melting temperatures at lower content of TWP in the composite. The melting and the crystallinity enthalpies as well as the crystallinity degree decrease with the incorporation of TWP in the composites. The thermal stability slightly increases with loadings of TWP up to 10 wt %. However, beyond the value of 10 wt % of TWP, the thermal properties decreased. The electrical resistivity of polymer composites decreased with increasing TWP and reached the percolation threshold (2.9 × 10^7^ Ohm.cm) at 10 wt % TWP, which was several orders of magnitude lower than that of the neat blend. This composite could have potential applications in different fields such as electronic materials and automotive industries. More studies on the mechanical and magnetic properties of the produced materials will be investigated in future work. 

## Figures and Tables

**Table 1:** Compositions of the composites low-density polyethylene/high impact polystyrene/ toner waste powder (LDPE/HIPS/TWP).

Samples	Composition of Polymer Blend		TWP wt % Relative to the Polymer Blend*
	LDPE (wt %)	HIPS (wt %)	
**C0**	80	20	0
C2	80	20	2
C6	80	20	6
C10	80	20	10
C15	80	20	15
C20	80	20	20

NB: *The percent weight of TWP is calculated according to Equation (1) based on the total weight of the blend polymer (LDPE/HIPS).

**Table 2:** The elementary composition of TWP at different positions by EDS analysis.

Elements % Atomic	Specter 1	Specter 2	Specter 3	Specter 4
C	70.9	72.6	73.7	23.3
O	21.4	20.5	18.9	41.6
Fe	7.4	6.2	6.7	1.4
Si	0.3	0.4	0.3	0.2
Ca	/	0.3	0.2	16.6
Ti	/	/	0.2	16.9

**Table 3:** Elemental composition of the TWP determined by X-ray fluorescence spectroscopy (XRF) analysis.

Main Elements	Percentage (%)	Trace Elements	Percentage (ppm)
Fe	71.43	Si	4973
Ti	3.02	Zn	4285
Ca	2.72	Mn	4117
Ce	1.73	P	1624

**Table 4:** Crystallization, melting temperatures and enthalpies, and degree of crystallinity, and electrical resistivity of LDPE/HIPS blend and LDPE/HIPS/TWP composites. The standard deviation is based on at least three measurements.

Sample	Melting		Crystallization		Crystallinity Degree	Electrical Resistivity
	T_m_ (°C)	ΔH_m_ (J/g)	Tc (°C)	ΔH_c_ (J/g)	χ_c_ (%)	Ohm cm
C0	112.1 ± 0.8	90.9 ± 0.6	96.6 ± 0.5	66.5 ± 0.8	25.4	7.37 × 10^14^
C2	113.4 ± 0.7	93.4 ± 0.6	94.6 ± 0.7	63.9 ± 0.5	24.4	2.36 × 10^13^
C6	113.3 ± 0.9	60.7 ± 0.7	97.3 ± 0.8	38.9 ± 0.8	15.4	2.56 × 10^9^
C10	114.5 ± 0.6	86.3 ± 0.8	98.0 ± 0.5	56.0 ± 0.6	24.1	2.90 × 10^7^
C15	112.0 ± 0.9	72.9 ± 1.1	95.3 ± 0.8	50.1 ± 0.7	22.6	2.77 × 10^7^
C20	110.0 ± 0.9	76.0 ± 0.9	97.5 ± 0.9	47.5 ± 1.0	18.01	2.70 × 10^7^

**Table 5:** Thermogravimetric results of polymer composites.

Samples	C0	C2	C6	C10	C15	C20
T (at 10% Mass loss) (°C)	417 ± 1	417 ± 1	418 ± 1	422 ± 1	411 ± 2	410 ± 2
T_max_ (°C)	462 ± 1	461 ± 1	462 ± 1	464 ± 1	459 ± 2	458 ± 2
Residue at 600 °C	0.01 ± 0.04	0.9 ± 0.1	2.3 ± 0.1	4.2 ± 0.1	6.1 ± 0.2	8.1 ± 0.2

## References

[1] Kabongo J.D., Idowu S.O., Capaldi N., Zu L., Gupta A.D. (2013). Waste Valorization. Encyclopedia of Corporate Social Responsibility.

[2] (2019). IMARC Report: Ink Market: Global Industry Trends, Share, Size, Growth, Opportunity and Forecast 2019–2024.

[3] Hassan T.A., Rangari V.K., Jeelani S. (2014). Value-Added Biopolymer Nanocomposites from Waste Eggshell-Based CaCO_3_ Nanoparticles as Fillers. ACS Sustain. Chem. Eng..

[4] Khalil H.A., Fizree H.M., Bhat A.H., Jawaid M., Abdullah C.K. (2013). Development and characterization of epoxy nanocomposites based on nano-structured oil palm ash. Compos. Part B Eng..

[5] Ismail H., Omar N.F., Othman N. (2011). Effect of carbon black loading on curing characteristics and mechanical properties of waste tyre dust/carbon black hybrid filler filled natural rubber compounds. J. Appl. Polym. Sci..

[6] Barhoum A., Shalan A.E., El-Hout S.I., Ali G.A.M., Abdelbasir S.M., Abu Serea E.S., Ibrahim A.H., Pal K. (2019). A Broad Family of Carbon Nanomaterials: Classification, Properties, Synthesis, and Emerging Applications. Handbook of Nanofibers.

[7] Barhoum A., Van Lokeren L., Rahier H., Dufresne A., Van Assche G. (2015). Roles of in Situ Surface Modification in Controlling the Growth and Crystallization of CaCO_3_ Nanoparticles, and Their Dispersion in Polymeric Materials. J. Mater. Sci..

[8] Essawy H.A., El-Sabbagh S.H., Tawfik M.E., Van Assche G., Barhoum A. (2017). Assessment of Provoked Compatibility of NBR/SBR Polymer Blend with Montmorillonite Amphiphiles from the Thermal Degradation Kinetics. Polym. Bull..

[9] Abdel-Haleem F.M., Saad M., Barhoum A., Bechelany M., Rizk M.S. (2018). PVC Membrane, Coated-Wire, and Carbon-Paste Ion-Selective Electrodes for Potentiometric Determination of Galantamine Hydrobromide. Physiological Fluids. Mater. Sci. Eng. C.

[10] Haichao L., Haoyi L., Bubakir M.M., Weimin Y., Barhoum A. (2018). Engineering Nanofibers as Electrode and Membrane Materials for Batteries, Supercapacitors, and Fuel Cells. Handbook of Nanofibers.

[11] Gopalakrishnan R., Li Y., Smekens J., Barhoum A., Van Assche G., Omar N., Van Mierlo J. (2019). Electrochemical Impedance Spectroscopy Characterization and Parameterization of Lithium Nickel Manganese Cobalt Oxide Pouch Cells: Dependency Analysis of Temperature and State of Charge. Ionics (Kiel)..

[12] Behera K., Yadav M., Chiu F.C., Rhee K.Y. (2019). Graphene Nanoplatelet-Reinforced Poly(vinylidene fluoride)/High Density Polyethylene Blend-Based Nanocomposites with Enhanced Thermal and Electrical Properties. Nanomaterials.

[13] Turky A.O., Barhoum A., Rashad M.M., Bechlany M. (2017). Enhanced the Structure and Optical Properties for ZnO/PVP Nanofibers Fabricated via Electrospinning Technique. J. Mater. Sci. Mater. Electron..

[14] Hammani S., Moulai-Mostefa N., Benyahia L. (2013). Effect of carbon black nanoparticle on the morphology rheology and thermal properties of emulsion polymer blends. Int. J. Nanotechnol..

[15] Cao L., Bai X., Lin Z., Zhang P., Deng S., Du X., Li W. (2017). The Preparation of Ag Nanoparticle and Ink Used for Inkjet Printing of Paper Based Conductive Patterns. Materials.

[16] Inglezakis V.J., Moustakas K. (2015). Household hazardous waste management: A review. J. Environ. Manag..

[17] Lu C., Zhang L., Zhong Y., Ren W., Tobias M., Mu Z., Ma Z., Geng Y., Xue B. (2015). An overview of e-waste management in China. J. Mater. Cycles Waste Manag..

[18] Rada E.C., Zatelli C., Cioca L.I., Torretta V. (2018). Selective Collection Quality Index for Municipal Solid Waste Management. Sustainability.

[19] Gecol H., Scamehorn J.F., Christian S.D., Grady B.P., Riddell F. (2001). Use of surfactants to remove water-based inks from plastics films. Colloids Surf. A Phys. Eng. Asp..

[20] Ruan J., Li J., Xu Z. (2011). An Environmental Friendly Recovery Production Line of Waste Toner Cartridges. J. Hazard. Mater..

[21] Vila A., Ferrer N., Garcia J.F. (2007). Chemical composition of contemporary black printing inks based on infrared spectroscopy: Basic information for the characterization and discrimination of artistic prints. Anal. Chim. Acta.

[22] Zayed M.A., Imam N.G., Ahmed M.A., El Sherbiny D.H. (2017). Spectrophotometric analysis of hematite/magnetite nanocomposites in comparison with EDX and XRF techniques. J. Mol. Liq..

[23] Barhoum A., García-Betancourt M.L., Rahier H., Van Assche G. (2018). Physicochemical Characterization of Nanomaterials: Polymorph, Composition, Wettability, and Thermal Stability. Emerging Applications of Nanoparticles and Architectural Nanostructures: Current Prospects and Future Trends.

[24] Barhoum A., Luisa García-Betancourt M. (2018). Physicochemical Characterization of Nanomaterials: Size, Morphology, Optical, Magnetic, and Electrical Properties. Emerging Applications of Nanoparticles and Architectural Nanostructures: Current Prospects and Future Trends.

[25] Zięba-Palus J., Kunicki M. (2006). Application of the micro-FTIR spectroscopy, Raman spectroscopy and XRF method examination of inks. Forensic Sci. Int..

[26] Kazeminezhad I., Mosivand S. (2014). Phase Transition of Electro oxidized Fe_3_O_4_ to γ and α-Fe_2_O_3_ Nanoparticles Using Sintering Treatment. Acta Phys. Pol. A.

[27] Chelliah M., Rayappan J.B.B., Krishnan U.M. (2012). Synthesis and characterisation of cerium oxide nanoparticles by hydoxide mediated approach. J. Appl. Sci..

[28] Zhao H., Fu W., Yang H., Zhao W., Chen Y.Z.H., Jing Q., Qi X., Cao J., Zhou X., Li Y. (2011). Synthesis and characterization of TiO_2_/Fe_2_O_3_ core-shell nanocomposition film and their photoelectron-chemical property. Appl. Surf. Sci..

[29] Sugimoto M., Shimada A., Kudoh H., Tamura K., Seguchi T. (2013). Product analysis for polyethylene degradation by radiation and thermal aging. Radiat. Phys. Chem..

[30] Ahangaran F., Hassanzadeh A., Nouri S., Neisiany R.E. (2017). Investigation of thermal and dielectric properties of Fe_3_O_4_/high-density polyethylene nanocomposites. J. Compos. Mater..

[31] Guo L., Gao S., An Q.-D., Xiao Z.-Y., Zhai S., Yang D., Cui L. (2019). Dopamine-derived cavities/Fe_3_O_4_ nanoparticles encapsulated carbonaceous composites with self generated three-dimensional network structure as an excellent microwave absorber. RSC Adv..

[32] Xu D., Wang P., Shen B. (2016). Development of TiO_2_-reduced graphene oxide nanocomposites and their enhanced photocatalytic and photovoltaic performance Digest. J. Nanomater. Biostruct..

[33] Nassar M.Y., Ali E.I., Zakaria E.S. (2017). Tunable auto-combustion preparation of TiO_2_ nanostructures as efficient adsorbents for the removal of an anionic textile dye. RSC Adv..

[34] Hu K., Cui Z., Yuan Y., Zhuang Q., Wang T., Liu X., Han Z. (2014). Synthesis, Structure, and Properties of High-Impact Polystyrene/Octavinyl Polyhedral Oligomeric Silsesquioxane Nanocomposites. Polym. Compos..

[35] Gaska K., Kádár R., Rybak A., Siwek A., Gubanski S. (2017). Gas Barrier, Thermal, Mechanical and Rheological Properties of Highly Aligned Graphene-LDPE Nanocomposites. Polymers.

[36] Hammani S., Barhoum A., Bechelany M. (2018). Fabrication of PMMA/ZnO Nanocomposite: Effect of High Nanoparticles Loading on the Optical and Thermal Properties. J. Mater. Sci..

[37] Samarth N.B., Mahanwar P.A. (2017). Study and characterization of LDPE/Polyolefin elastomer and LDPE/EPDM blend: Effect of chlorinated water on blend performance. Adv. Manuf. Polym. Compos. Sci..

[38] Zheng L., Li Y., Weng Y., Zhu J., Zeng J. (2019). Localization control of carbon nanotubes in immiscible polymer blends through dynamic vulcanization. Compos. Part B.

[39] Tao F., Nysten B., Baudouin A., Thomassin J., Vuluga D., Detrembleur C., Bailly C. (2011). Influence of nanoparticle-polymer interactions on the apparent migration behaviour of carbon nanotubes in an immiscible polymer blend. Polymer.

[40] Kausar A., Taherian R. (2019). Electrical Conductivity in Polymer Composite Filled With Carbon Microfillers. Electrical Conductivity in Polymer-Based Composites: Experiments, Modelling and Applications.

[41] Liu Z., Peng W., Zare Y., Hui D., Rhee K.Y. (2018). Predicting the Electrical Conductivity in Polymer Carbon Nanotube Nanocomposites Based on the Volume Fractions and Resistances of the Nanoparticle, Interphase, and Tunneling Regions in Conductive Networks. RSC Adv..

[42] Kyrylyuk A.V., van der Schoot P. (2008). Continuum percolation of carbon nanotubes in polymeric and colloidal media. Proc. Natl. Acad. Sci. USA.

[43] Otten R.H., van der Schoot P. (2009). Continuum Percolation of Polydisperse Nanofillers. Phys. Rev. Lett..

[44] Luyt A.S., Molefi J.A., Krump H. (2006). Thermal, mechanical and electrical properties of copper powder filled low-density and linear low-density polyethylene composites. Polym. Degrad. Stab..

